# Engineering and Structural Elucidation of a Sac7d‐Derived IgG Fc‐Specific Affitin and Its Application for the Light‐Controlled Affinity Purification of Antibodies

**DOI:** 10.1002/cbic.202500102

**Published:** 2025-04-07

**Authors:** Felix Veitl, Andreas Eichinger, Peter Mayrhofer, Markus R. Anneser, Mauricio Testanera, Kilian Rauscher, Matthias Lenz, Arne Skerra

**Affiliations:** ^1^ Chair of Biological Chemistry School of Life Sciences Technical University of Munich 85354 Freising Germany

**Keywords:** affinity chromatography, affitin, antibody, Fc fragment, protein engineering

## Abstract

While protein A affinity chromatography is widely established for antibody purification, the acidic elution conditions often lead to protein aggregation and deamidation. Here, an alternative approach is described for the purification of antibodies utilizing an engineered binding protein based on the archaebacterial Sac7d scaffold in combination with light‐controlled α‐CD affinity chromatography (Excitography). Starting from a published affitin molecule, a monomeric protein version (C3A24) was engineered by substituting the unpaired thiol side chain Cys24 within the binding site by Ala, and, unexpectedly, its binding activity towards the human IgG1 Fc region was even improved (*K*_D_ = 76 nM). X‐ray analysis of the cocrystallized C3A24 with a recombinant human Fc fragment revealed a 2:1 stoichiometry, with a binding site at the junction between the C_H_2 and C_H_3 domains. Interestingly, this binding site coincides with the ones of protein A, protein G, and the neonatal Fc receptor (FcRn). The affitin/Fc interaction is dominated by a network of hydrogen bonds, whereas, unpredicted by the initial affitin design, the two C‐terminal Lys residues are also involved via a salt bridge and another hydrogen bond. Using the Azo‐tagged C3A24, we purified clinically relevant antibodies from cell culture medium in a single step under physiological buffer conditions.

## Graphical abstract

An affitin derived from the Sac7d scaffold enables the light‐controlled affinity purification of antibodies under physiological conditions, thus overcoming limitations of protein A chromatography. X‐ray crystallographic analysis and affinity measurements reveal complex formation with Fc at the C_H_2/C_H_3 junction, similar to protein A/G and FnRn. Combined with the light‐responsive Azo‐tag, this tiny adapter molecule opens new applications in antibody research.

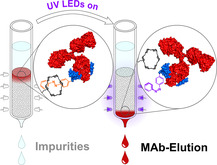

## 1 Introduction

The development of the pharmaceutical landscape during the last two decades has revealed a clear shift from small molecule drugs toward biologics, in particular monoclonal (or recombinant) antibodies (mAbs) as well as Fc‐fusion proteins. Since the approval of the first therapeutic antibody, muromonab‐CD3, by the U.S. Food and Drug Administration in 1986 and also the subsequent approval of the first recombinant humanized antibody, trastuzumab, in 1998^[^ ^1^ ^]^ the number of mAbs in clinical use has grown exponentially, accounting for more than half of the top ten therapeutics according to global sales.^[^ ^2^ ^]^ This underscores the crucial need for efficient and cost‐effective manufacturing processes, including potent purification techniques. In addition, there is ongoing demand for the quick isolation of antibodies at small scale in biomedical research.

In both areas, protein A affinity chromatography remains the gold standard for mAb purification due to its high selectivity and robust performance,^[^ ^3^ ^]^ also under conditions of Good Manufacturing Practice (GMP). Natural protein A is a membrane protein of the pathogenic bacterium *Staphylococcus aureus* ^[^ ^4^ ^]^ comprising five immunoglobulin (Ig)‐binding domains.^[^ ^5^ ^]^ In its natural context, protein A supports evasion from the humoral immune response as it both mediates decoration of the bacterial surface with plasma Ig without triggering effector functions and blocks antibody activity via its soluble fragments.^[^ ^6^ ^]^

The coding sequence of protein A was already elucidated in the early days of recombinant DNA technology,^[^ ^7^ ^]^ which allowed the engineering of improved affinity reagents based on the B domain, or the 58‐residue synthetic consensus Z domain, both comprising a three‐α‐helix bundle fold.^[^ ^8^ ^]^ Most protein A chromatography resins on the market still utilize either the full‐length recombinant protein or modified versions constructed from four to six repeats of the B or C domain^[^ ^9^ ^]^ and apply an acidic pH shift to elute the bound antibody. Albeit state of the art, the performance of this technology is hampered by the high cost for the affinity matrix^[^ ^10^ ^]^ and the stressful purification conditions for the antibody, promoting aggregation and deamidation of Asn residues, for example.^[^ ^11^ ^]^

Driven by the drawbacks of protein A, optimized variants have been developed which, however, do not fully abolish the shortcomings of this technology.^[^ ^9^ ^]^ In the search for more promising alternative affinity ligands useful for mAb affinity purification, other bacterial Ig receptors have attracted attention, such as protein G from group G *Streptococci* ^[^ ^12^ ^]^ and protein L from *Peptostreptococcus magnus*.^[^ ^13^ ^]^ Furthermore, engineered protein scaffolds, in particular the Z domain as well as affibodies derived from protein A, have been proposed as new tools for antibody purification.^[^ ^14^ ^]^ More recently, a suite of different affinity ligands based on single‐domain antibody fragments from camelids (VHH) has been commercially developed as CaptureSelect affinity matrix.^[^ ^15^ ^]^

Beyond that, a more exotic group of binding proteins directed against the Fc portion of antibodies was engineered based on a small (7 kDa) DNA‐binding protein, Sac7d, from the thermoacidophilic archaebacterium *Sulfolobus acidocaldarius* or related proteins, such as Sso7d from *S. solfataricus*.^[^ ^16^ ^]^ Over the years, a series of so‐called affitins (later also dubbed nanofitins) were generated as artificial binding reagents towards diverse protein targets using focused random mutagenesis and in vitro selection via yeast or ribosome display. Specifically, three affitins were selected against the human IgG Fc and biochemically characterized: Sac7d‐C3 and Sac7d‐D1^[^ ^16^ ^]^ as well as Sso7d‐hFc.^[^ ^17^ ^]^

Affitins offer certain benefits as antibody mimetics^[^ ^18^ ^]^ due to their small size and robust fold, comprising a single polypeptide chain of just 66 amino acids, which forms five β‐strands and a C‐terminal α‐helix, while lacking disulfide bridges and posttranslational modifications.^[^ ^19^ ^]^ In these biochemical properties, the engineered affitins closely resemble the B or Z domain of protein A, in spite of the differing protein fold. Indeed, the affitin Sac7d‐D1 was successfully used as an affinity ligand and immobilized to a chromatography matrix in order to purify human IgG1 antibodies,^[^ ^20^ ^]^ again using an acid elution step. However, no other published examples have followed this proof‐of‐concept study, and further optimization by protein engineering was hampered by the lack of structural information on the mode of complex formation between this affitin and the Ig Fc fragment.

When we compared the three anti‐Fc affitins mentioned above in preliminary experiments—using *E. coli* surface display^[^ ^21^ ^]^—only Sac7d‐C3 exhibited robust binding activity towards the prototypic humanized IgG1 antibody trastuzumab,^[^ ^22^ ^]^ thus emerging as the most promising candidate for further study. To gain understanding of its binding mechanism and dynamics, we have now elucidated the structure of Sac7d‐C3 in complex with a recombinant human IgG1 Fc fragment (recFc) using X‐ray crystallography. Based on these findings, we propose a novel application of an improved Sac7d‐derived IgG binding protein, C3A24, for the light‐controlled affinity purification of antibodies using the recently developed Azo‐tag.^[^ ^23^ ^]^

## 2 Results and Discussion

### 2.1 Structure Determination of the Complex Between the Ig Fc Fragment and a Monomeric Affitin

The affitin Sac7d‐C3^[^ ^16^ ^]^ was produced in the cytoplasm of *E. coli* as a maltose‐binding protein (MBP) fusion protein, from which it was liberated by proteolytic cleavage, followed by subtractive immobilized metal ion affinity chromatography (IMAC) and, if necessary, size exclusion chromatography (SEC). During initial expression tests, a varying degree of dimerization was observed for the affitin C3 via non‐reduced SDS‐PAGE, which was likely caused by interchain disulfide bond formation between the unpaired Cys24 residues in two protein molecules. While a tendency to form covalent dimers was already noted in the original publication,^[^ ^16^ ^]^ the replacement of residue Cys24 by Ser abolished dimer formation and permitted crystallization of the uncomplexed affitin (PDB ID: 2XIW), but this amino acid exchange also led to a considerably diminished affinity towards IgG (from *K*_D_ = 74 nM to about 223 nM).

To further investigate this aspect, we created protein variants carrying a short N‐terminal Azo‐tag (Gly‐Pap‐Gly‐Pro, see below), thus allowing the one‐step purification—in the absence of a fusion partner—controlled by mild UV light.^[^ ^23^ ^]^ Although in our hands the *K*_D_ values of both Azo‐C3 and Azo‐C3(C24S) versus trastuzumab measured by real‐time surface plasmon resonance (SPR) spectroscopy were slightly different from the published values,^[^ ^16^ ^]^ replacement of Cys24 with Ala resulted in an even better *K*_D_ value of 76 ± 4.0 nM (see further below). This indicated that the influence of residue Cys24 on the binding activity towards the Ig Fc region might not be as significant as previously assumed, and the mutation Cys24 → Ala to stabilize the monomeric affitin is even beneficial, thus leading to the improved mutant C3A24.

To investigate the binding mechanism of C3A24 towards the human IgG1 Fc fragment, the three‐dimensional structure of the complex between C3A24 and recFc was elucidated by X‐ray crystallography (**Table** **1**). RecFc was produced in the less‐reducing cytoplasm of *E. coli* Origami^[^ ^24^ ^]^ and purified as a non‐glycosylated homodimer using IMAC and cation exchange chromatography (CEX). For the cocrystallization experiments, C3A24 was produced as a fusion protein with N‐terminal MBP, which was afterwards removed by cleavage with Tobacco Etch Virus (TEV) protease. Due to the cloning procedure for the affitin, the resulting small protein (residues 2–66) additionally carried the short sequence DAEF at its N‐terminus. Based on the hypothesis that two affitin molecules bind to one homodimeric Fc fragment, both purified binding partners were mixed at a ratio of 4:1 (referring to the individual polypeptide chains). The excess of the small affitin ligand ensured complete saturation of the binding sites on the Fc fragment. After overnight incubation at room temperature in 150 mM Tris/HCl at pH 7.5, the complex was isolated via SEC (see Figure S1, Supporting Information).

**Table 1:** Crystallographic analysis and refinement statistics.

	C3A24•recFc
Crystal data:	
Space group	P2_1_
Unit cell dimensions: *a*, *b*, *c* [Å] *α*, *β*, *γ* [°]	68.3, 136.8, 69.8 90.0, 90.1, 90.0
Molecules per a.u.	4 Fc chains + 4 affitin chains
Data collection:	
Wavelength [Å]	0.97620
Resolution range [Å]^a)^	69.76–2.90 (3.00–2.90)
I/*σ*[I]a)	7.7 (1.5)
*R*_meas_ [%]a), b)	11.9 (90.2)
Unique reflections	27 577
Multiplicitya)	3.1 (3.1)
Completenessa)	97.0 (98.7)
Refinement:	
*R*_cryst_/*R*_free_ b)	22.7/27.9
Protein atoms	8892
Average B‐factor [Å^2^]	88.3
Geometry:	
R.m.s.d. bond lengths, angles [Å, °]	0.006, 1.443
Ramachandran analysisc): Core, allowed, generously allowed, disallowed [%]	87.8, 11.7, 0.5, 0.0

a) Values in parentheses are for the highest resolution shell;

b) *R*_meas_, *R*_cryst_, and *R*_free_ according to Diederichs & Karplus^[^ ^64^ ^]^ and Wilson;^[^ ^65^ ^]^

c) calculated with PROCHECK.

### 2.2 Structure of the Affitin C3A24 in Complex with a Human IgG1 Fc Fragment

**Figure 1:**
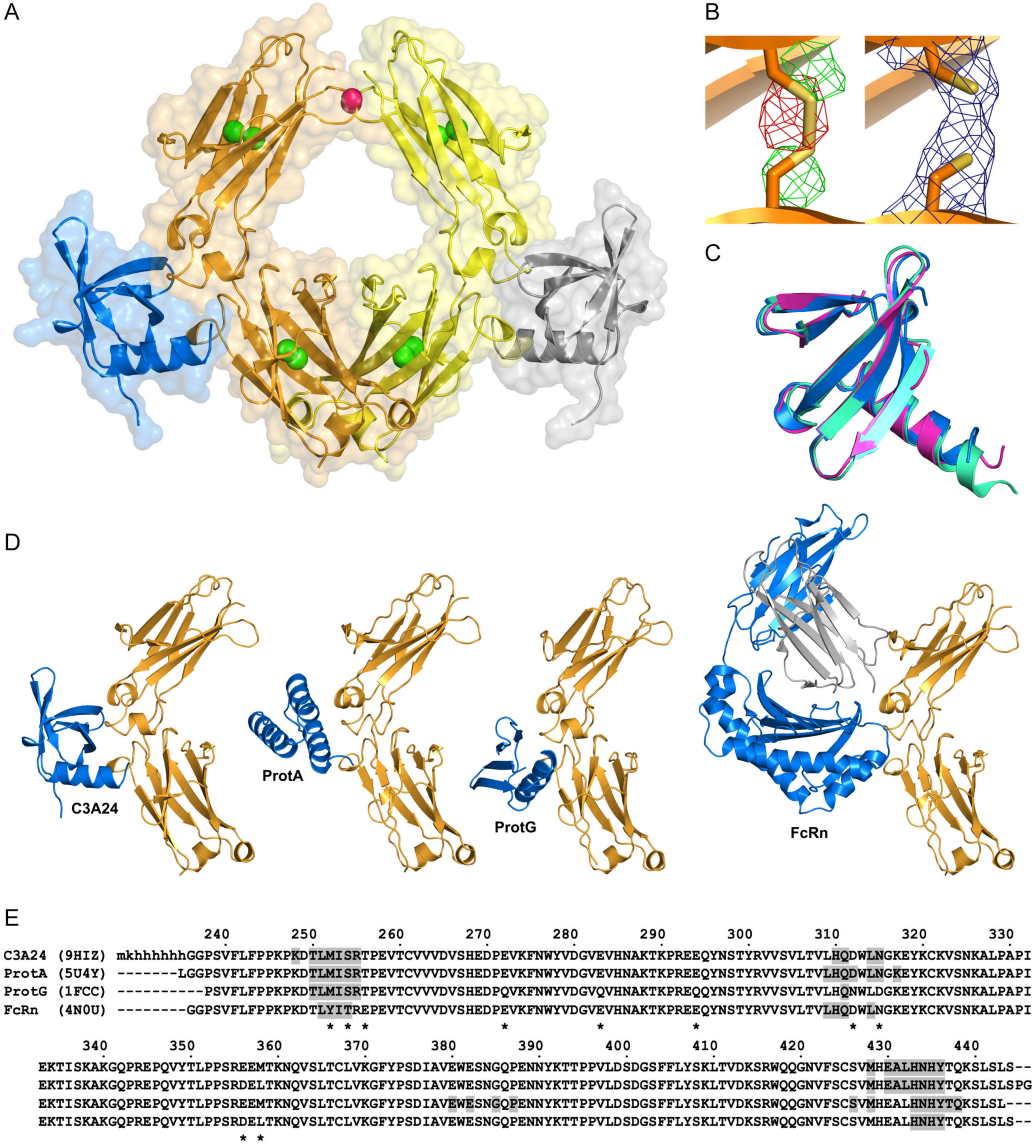
Crystal structure of the affitin C3A24 in complex with a recombinant human IgG1 Fc fragment (recFc). A) The complex consists of two Ig heavy chain fragments (chain A: orange; chain B: yellow), which are associated as a homodimer to form the Fc fragment. One affitin molecule (marine/gray) binds to each heavy chain in the contact region between the C_H_2 and C_H_3 domains. Conserved Cys residues located within the Ig β‐sandwich are shown as spheres (green), whereas the glycosylation site Asn297 of natural human IgG1 is highlighted in purple. B) Close‐up view of the Cys residues 367 and 425 within C_H_3 of the Fc fragment (chain D in the a.u.). The Cys residues of the finally refined structure (right) and, for comparison, a molecular model refined with constrained disulfide bonds (left) are shown as sticks, while the polypeptide backbone is depicted as a cartoon. The 2F_O_‐F_C_ electron density is contoured at 1σ in dark blue; positive F_O_‐F_C_ density is colored in green, and negative density is colored red (both at 2σ). C) Superposition of C3A24 (marine) with Sac7d (purple) (PDB ID: 1AZP) and C3(C24S) (cyan) (PDB ID: 2XIW). D) Comparison of the crystal structures of the affitin C3A24 in complex with recFc (PDB ID: 9HIZ) with the B domain of protein A in complex with IgG1 Fc (PDB ID: 5U4Y), protein G in complex with IgG1 Fc (PDB ID: 1FCC), and FcRn in complex with IgG1 Fc (PDB ID: 4N0U). While protein A comprises a bundle of three α‐helices, the affitin forms a 5‐stranded β‐sheet with a C‐terminal α‐helix, somewhat similar to the tertiary structure of protein G but associating with Fc in a different orientation. The FcRn has an entirely different tertiary structure, including the paired β_2_m domain (gray). E) Sequence alignment between recFc and the IgG1 Fc fragments from the complexes depicted in (C) (PDB IDs: 5U4Y, 1FCC, and 4N0U). Amino acid residues in Fc contacting the affitin C3A24, protein A, protein G, or the FcRn, respectively, as determined by PISA analysis, are highlighted in gray. Asterisks indicate some variations in the Ig sequences.

In the crystal structure, two Ig heavy chains are associated to form an intact Fc fragment (**Figure** **1**), despite the absence of the hinge region in the recombinant protein, which in the intact IgG1 provides crosslinks between the paired heavy chain regions via two disulfide bridges.^[^ ^25^ ^]^ Interestingly, the pairwise residues Cys261 and Cys321, as well as Cys367 and Cys425, respectively, in the Fc chain, which normally give rise to the conserved disulfide bridges within each β‐sandwich of the Ig domains C_H_2 and C_H_3, all appeared to be present in their reduced thiol form (for both Fc molecules in the asymmetric unit, a.u.). This became apparent from the large distances between the sulfur atoms of the corresponding pairs of thiol side chains—3.5 Å in the C_H_2 and 3.4 Å in the C_H_3 domain—as evident from the electron density map (Figure 1B). On the other hand, when the crystal structure was refined with constraints for the corresponding disulfide bonds, negative electron density appeared between the sulfur atoms.

Furthermore, mass spectrometric (MS) analysis corroborated the absence of disulfide bonds in at least 50% of the recFc molecules, with a measured value of 24,743 Da for the Fc chain, i.e., 4 Da larger than expected for the fully oxidized polypeptide (see Figure S2, Supporting Information). Considering that *E. coli* Origami B was genetically engineered to facilitate the formation of disulfide bonds in its less‐reducing cytoplasm,^[^ ^24^ ^]^ and that the production of functional antibody fragments in this strain was described before,^[^ ^26^ ^]^ this was unexpected. However, the structurally uncritical role of the central disulfide bridge has been documented for variable Ig domains,^[^ ^27^ ^]^ and the absence of disulfide bonds in intrabodies functionally expressed in the cytoplasm of eukaryotic cells was also reported.^[^ ^28^ ^]^ These previous findings are in line with the correct folding of the C_H_2 and C_H_3 domains of the recFc and the proper association of its two heavy chains as seen in the crystal structure solved here (Figure 1A). In fact, a superposition of the 206 Cα atoms of residues 238–443 in chain A with the corresponding atom positions of the crystallized α2,6‐sialylated Fc fragment derived from human IgG1, which has all disulfide bonds properly formed (PDB ID: 4CDH),^[^ ^29^ ^]^ resulted in a very low RMSD of 1.22 Å, thus confirming the absence of relevant structural deviations for recFc.

In the present crystal structure, the homodimeric Fc fragment appeared tightly associated with two molecules of the optimized affitin C3A24, which both bind to the linker region connecting its C_H_2 and C_H_3 domains (Figure 1A). The symmetry‐related interface of each affitin molecule is formed by three β‐strands (β3, β4, and β5), consistent with the positional randomization strategy that led to the selection of Sac7d‐C3^[^ ^16^ ^]^—and also in agreement with some previously published or crystallized affitins directed against PulD,^[^ ^30^ ^]^ CelD, or lysozyme^[^ ^31^ ^]^ as well as the green fluorescent protein, GFP.^[^ ^32^ ^]^

To compare the structure of the engineered affitin C3A24 in the complex with recFc with the X‐ray structures of the wildtype scaffold Sac7d (PDB ID: 1AZP)^[^ ^33^ ^]^ and the individually crystallized mutated affitin C3(C24S) (PDB ID: 2XIW),^[^ ^16^ ^]^ the proteins were superimposed (Figure 1C). All three Sac7d variants were highly similar, with a root mean square deviation (RMSD) = 1.23 Å between C3A24 and Sac7d and RMSD = 1.13 Å between C3A24 and C3(C24S), for 65 pairwise superimposed Cα‐atoms. This is remarkable with respect to Sac7d, which differs in 15 amino acids from C3A24 and does not bind IgG, thus confirming the high tolerance of the Sac7d β‐sheet towards side chain replacements.^[^ ^16^, ^31^ ^]^ Although C3(C24S) differs in merely one amino acid from C3A24, its RMSD is larger, which might indicate a small conformational adaptation of the latter upon complex formation with recFc. However, these differences are mainly confined to the loop regions, while the conformation of the binding interface, which is dominated by the three β‐strands (β3, β4, β5), is almost identical in all three proteins.

To further examine the contact region and the identity of the surface residues in the Fc fragment that participate in the interactions, we conducted a PISA analysis^[^ ^34^ ^]^ of the affitin·recFc complex. For comparison, the same kind of analysis was performed with crystal structures of some other well‐known binding proteins for the IgG1 Fc region: protein A (PDB ID: 5U4Y),^[^ ^35^ ^]^ protein G (PDB ID: 1FCC)^[12a]^ and the neonatal Fc receptor (FcRn) (PDB ID: 4N0U).^[^ ^36^ ^]^ Surprisingly, it appeared that the affitin binding site almost entirely overlaps with those of protein A, protein G, and FcRn (Figure 1D). In spite of the different tertiary structures of protein A, which is an α‐helix bundle, and C3A24, with its β‐sheet interface—similar to protein G (Figure 1C)—the Fc residues involved in these interactions are almost identical and form three distinct clusters in its amino acid sequence (Figure 1E). Notably, FcRn with its completely different protein architecture essentially shares the same binding site, which is critical for the long IgG plasma half‐life through endosomal recycling^[^ ^37^ ^]^ and also for the charging of MHC class I and II and antigen presentation.^[^ ^38^ ^]^ Hence, the elbow region connecting the IgG C_H_2 and C_H_3 domains seems to be a focus point for protein–protein interactions.

**Figure 2:**
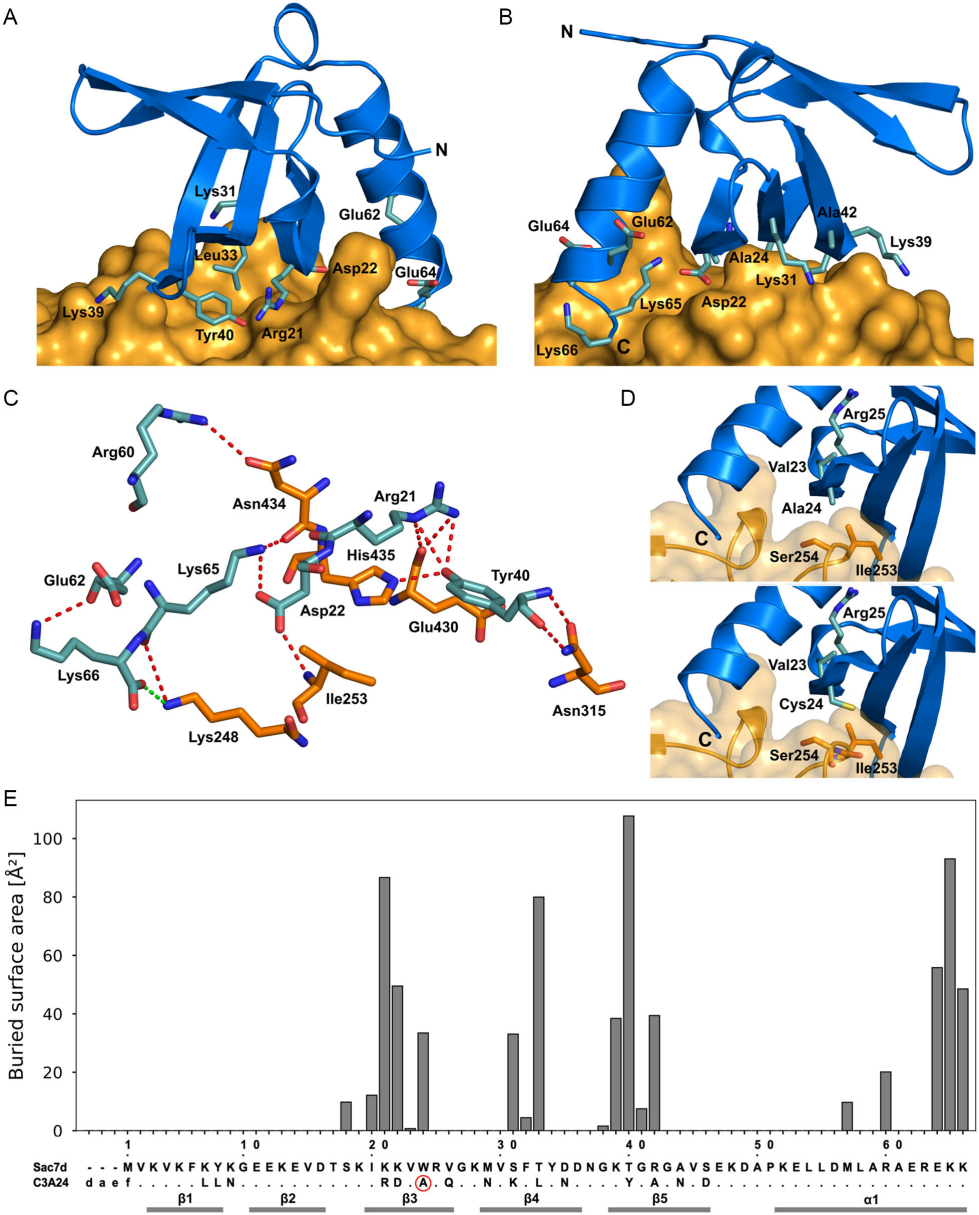
Detailed analysis of the interactions that stabilize the affitin•recFc complex. A) Interface between recFc (depicted with its surface in orange) and C3A24 (marine cartoon with relevant side chains shown as sticks). B) Same as (A) viewed from the back. C) The hydrogen‐bonding network between the affitin and recFc is depicted with red dashed lines (carbon atoms in the affitin colored light teal). The C‐terminal carboxylate group of Lys66 in C3A24 appears to form a salt bridge (green) with Lys248 in recFc. D) Top: Close‐up view of Ala24 in C3A24 (marine and light teal) and the surrounding Fc residues (orange). Bottom: Ala replaced by Cys using PyMOL software. E) PISA analysis to determine the BSA in the complex with recFc for each residue of the affitin. An amino acid sequence alignment between C3A24 (position Ala24 highlighted with a red circle) and the native protein scaffold, Sac7d, with secondary structure elements (as defined on the basis of the present crystal structure) indicated underneath.

Closer investigation of the interface with the Fc fragment on the side of the affitin further revealed the involvement of several residues in its strands β3, β4, and β5: Arg21, Asp22, Ala24, Lys31, Leu33, Tyr40, and Ala42 (**Figure** **2**). All of these residues had been mutated in Sac7d to generate C3.^[^ ^16^ ^]^ Conversely, 8 of the 15 positions that were randomized in this scaffold to prepare the affitin library do not form contacts with the Fc fragment. Calculation of the buried surface area (BSA) between the affitin and the Fc fragment revealed four distinct clusters of interacting residues on C3A24 (Figure 2E). While clusters #1 to #3 correspond to the three β‐strands mentioned above, cluster #4 comprises the C‐terminal residues Glu64, Lys65 and Lys66, which are located at the end of the α‐helix that packs against the β‐sheet of Sac7d.

Overall, the interaction between the affitin and the Fc fragment is stabilized by a network of hydrogen bonds that arise from six residues in the engineered binding protein (Figure 2C). Among those, Tyr40 makes the most significant contributions: both its backbone carbonyl oxygen and amide nitrogen form hydrogen bonds with the side chain carboxamide group of Asn315 in recFc, while its phenolic hydroxyl group forms a hydrogen bond with the main chain amide group of His435. Furthermore, the guanidinium group of Arg21 gives rise to two hydrogen bonds with the main chain carbonyl oxygen of Glu430 in recFc. Finally, the carboxylate group of Asp22 forms a hydrogen bond interaction with the backbone amide nitrogen of Ile253, and the guanidinium group of Arg60 is engaged in a hydrogen bond with the side chain carbonyl oxygen of Asn434.

Notably, the N‐terminus of the affitin, including the structurally flexible DAEF extension, is not involved in complex formation and faces the solvent. This should allow regioselective coupling to an affinity matrix or construction of fusion proteins via the N‐terminus without influencing the Fc‐binding activity. On the other hand, the C‐terminal residues of C3A24 are involved in dense contacts with recFc. This observation contrasts with published data claiming that, in general, none of the affitin polypeptide termini is involved in interactions with their respective complex partners.^[^ ^32^ ^]^ In the present case, however, Lys65 of C3A24 forms two hydrogen bonds, one via its main chain carbonyl oxygen with the ε‐amino group of Lys248 in the Fc fragment and another one via its ε‐amino group with the backbone carbonyl oxygen of Asn434 there. Furthermore, it seems that the tentatively modeled residue Lys66, despite its low electron density, contributes a salt bridge via its C‐terminal carboxylate group with the ε‐amino group of Lys248 in recFc. By also forming an intramolecular hydrogen bond between its ε‐amino group and the carbonyl oxygen of Glu62 in its vicinity, Lys66 may even play a dual role in stabilizing the complex (Figure 2C). However, its poor structural definition in the electron density map indicates enhanced structural flexibility of this C‐terminal residue, suggesting that Lys66 energetically contributes mainly via less directed electrostatic effects. In fact, the Lys66 side chain could alternatively adopt a slightly different orientation, allowing the formation of a hydrogen bond with the main chain carbonyl oxygen of Gly385 in recFc.

In light of the published lower affinity of the previously described mutant C3(C24S)^[^ ^16^ ^]^ compared to C3A24, we investigated the molecular surroundings of Ala24 in the complex with recFc (Figure 2D). Indeed, the small Ala side chain snugly fits between the two nearest residues in recFc, Ile253, and Ser254. Notably, when Ala24 was changed to Cys using PyMOL molecular modeling software, all possible rotamers led to sterical clashes with surrounding side chains, without indication for additional polar interactions. This suggests either a conformational change, where neighboring residues move in order to provide space for the larger Cys side chain, or a slightly differing mode of complex formation for the original affitin C3. Due to the hydroxyl group of Ser, sterical clashes also have to be expected in the case of the mutant C3(C24S), apart from the stronger solvation tendency of its more polar side chain. Interestingly, when we revisited the affinity towards Fc of the affitin mutants in question using the same molecular format (see below), it turned out that C3A24 actually showed a two‐fold lower *K*_D_ value, whereas the affinities of the Cys24 and the Ser24 versions were indistinguishable (**Table** **2**), which nicely matches these structural observations.

**Table 2:** Affinities of (azo‐tagged) C3 variants towards IgG1 Fc determined by SPR.

Protein	*K*_D_ [nM]a)
C3b)	161 ± 2.4
C3(C24S)	173 ± 5.3
C3(C24A)	76 ± 4.0
C3A24(ΔK66)	212 ± 8.5
C3A24(K66A)	177 ± 6.1

a) Mean steady‐state *K*_D_ calculated for three separate measurements;

b) Protein was kept in the presence of 5 mM dithiothreitol (DTT) until measurement.

### 2.3 Role of the C‐Terminal Lys Residue on the Interaction Between the Affitin and the Fc Fragment

To elucidate the influence of the C‐terminal affitin residue on the affinity to the Fc fragment, Lys66 was either replaced by Ala or deleted using site‐directed mutagenesis. Corresponding mutants in the form of the Azo‐C3A24 protein were expressed in *E. coli* NEBExpress(lowRF1) and purified via light‐controlled α‐CD affinity chromatography, along with the parental Azo‐tagged protein. Real‐time SPR measurements were then performed in triplicates using trastuzumab immobilized onto the sensor chip (Table 2).

Notably, fitting the data with a simple 1:1 Langmuir binding model resulted in a poor match, with a significant discrepancy between the curve fit and the observed ascending trend in the baseline for all mutants investigated (see Figure S3, Supporting Information). However, applying a 1:1 binding model that also accounts for a secondary conformational change (i.e., a two‐state reaction model) resolved this discrepancy for all affitin versions. Together with the fact that an extended incubation of the affitin and the Fc fragment appeared to be beneficial to isolate the recFc·C3A24 complex via SEC at high yield for protein crystallization, this indicates a conformational change after the fast initial binding event (see Figure S3, Supporting Information). Therefore, to allow a more reliable determination of the *K*_D_ values, the SPR data were fitted at equilibrium using a steady‐state model (Table 2).

As anticipated from the tight complex formation seen in the crystal structure (Figure 1A), the deletion of Lys66 led to an almost 3‐fold reduction in affinity, with *K*_D_ = 212 ± 8.5 nM, compared to *K*_D_ = 76 ± 4.0 nM for the reference protein, Azo‐C3A24. Moreover, the binding kinetics changed to a decreased *k*_on_ and an increased *k*_off_ rate, rendering the affitin‐Fc interaction significantly more dynamic (see Table S1, Supporting Information). Similar results, albeit less pronounced than for Azo‐C3A24(ΔK66), were obtained for the mutant Lys66 → Ala, with *K*_D_ = 177 ± 6.1 nM. Thus, the loss of the positive charge in the side chain alone resulted in a comparable affinity reduction as the full deletion of Lys66. This indicates that the salt bridge formed by the terminal carboxylate group of the affitin to Lys248 in the Fc fragment is less relevant for the binding activity than the stabilization by the side chain of Lys66 via an H‐bond to Glu62 within the affitin (Figure 2C). On the other hand, considering the poor definition of Lys66 in the electron density map, one may also speculate that the loss of the latter intramolecular interaction by means of the Lys66 → Ala substitution further enhances the flexibility of the C‐terminal residue such that the involvement of its carboxylate group in the intermolecular salt bridge with the Fc fragment is no longer energetically relevant due to an entropic effect.

### 2.4 Application of the Azo‐Tagged Engineered Affitin for the Light‐Controlled One‐Step Purification of Antibodies

Up to now, all affinity purification protocols for antibodies using specific binding proteins rely on an acidic elution step to dissociate the bound Ig protein from the chromatography column. This is widely known and well established in particular for protein A and G affinity chromatography^[^ ^39^ ^]^ but also for the CaptureSelect affinity matrix.^[^ ^15^ ^]^ Similarly, a 100 mM glycine/HCl buffer at pH 2.5 was previously applied when using the affitin Sso7d‐D1 for antibody affinity purification.^[^ ^20^ ^]^ With the goal of avoiding such potentially harmful harsh conditions, we used the recently described Azo‐tag^[^ ^23^ ^]^ to create a small affitin adapter molecule that allows the one‐step purification of antibodies on a robust α‐CD affinity column simply controlled by light and, notably, in a mild buffer of choice. The Azo‐tag comprises the light‐switchable non‐canonical amino acid p‐(phenylazo)‐L‐phenylalanine (Pap), which can be efficiently incorporated using an expanded genetic code via amber stop codon suppression and was already applied above. For the present application, we produced the stabilized monomeric affitin C3A24 with an N‐terminal Azo‐tag (Gly‐Pap‐Gly‐Pro, lacking the DAEF extension) using the improved expression vector pSB22 and *E. coli* NEBExpress(lowRF1) cells.^[^ ^23^ ^]^

**Figure 3:**
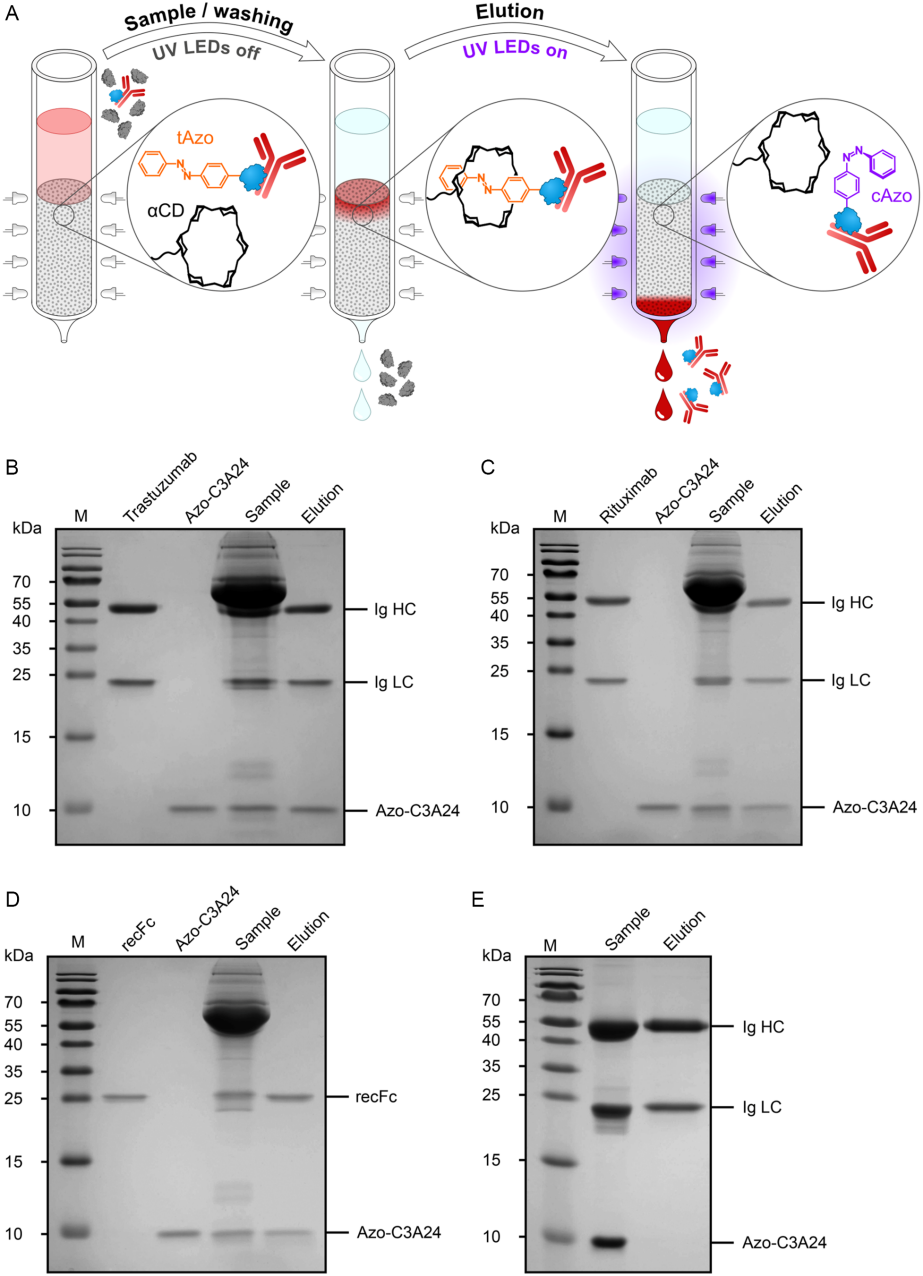
Purification of antibodies using the Azo‐tagged affitin via light‐controlled α‐CD affinity chromatography. A) Schematic illustration of the principle of Excitography. The sample containing the antibody in the cell culture medium is mixed with the Azo‐C3A24 adapter molecule and applied to the α‐CD affinity column. With the Pap residue initially in the trans‐configuration, the Azo‐tag binds to the chromatography matrix by non‐covalent complex formation with the immobilized α‐CD groups, thus specifically capturing the antibody·affitin complex. After washing away all impurities, the elution of the antibody complex is triggered by illumination with UV light at 355 nm based on the configurational switch to cis‐Pap. B,C) Demonstration of the one‐step purification of different Ig molecules from cell culture medium containing 5% v/v FCS via SDS‐PAGE analysis: (B) trastuzumab, (C) rituximab, and D) the recFc fragment. Lanes: 1, PageRuler Prestained Protein Ladder; 2, the isolated Ig protein for comparison; 3, the purified Azo‐C3A24 adapter molecule; 4, DMEM/FCS spiked with the Ig protein; and 5, the purified Ig protein that was eluted, together with the Azo‐C3A24 adapter molecule, from the α‐CD affinity column upon exposure to mild UV light using a physiological running buffer (50 mM Tris/HCl pH 7.5, 150 mM NaCl). E) The Azo‐affitin adapter protein can be easily removed from the purified antibody solution via SEC in the presence of 1.5 M urea in α‐CD buffer, after a short incubation for dissociation, as documented by SDS‐PAGE. Lanes: 1, Prestained Protein Ladder; 2, sample of purified trastuzumab in complex with Azo‐C3A24; 3, eluate from SEC.

To prepare the adapter protein, Azo‐C3A24, a lysate of the transformed bacteria was applied to the α‐CD affinity column (1 mL bed volume), and, after washing with 2 mL of a physiological buffer, the bound affitin was eluted via exposure to mild UV light at 355 nm from a panel of LEDs.^[^ ^23^ ^]^ The purified Azo‐C3A24 was subsequently added to DMEM as a typical cell culture medium, which was supplemented with 5% v/v fetal calf serum (FCS) and spiked with different Ig proteins, using a 4:1 stoichiometric ratio: trastuzumab,^[^ ^22^ ^]^ rituximab,^[^ ^40^ ^]^ or recFc. After incubation at room temperature for 10 min (or overnight), the mixture was applied to a fresh α‐CD affinity column, washed with 3 mL α‐CD buffer, and the affitin·antibody complex was eluted in the same buffer at physiological pH by illuminating the column with 355 nm UV light (**Figure** **3**). While trastuzumab and rituximab, as well as the recombinant Fc fragment, constituted only a minor proportion of the total protein in the loaded sample, which was dominated by the strong band for bovine serum albumin (BSA) from the FCS, the eluted protein was highly pure, comprising only the immunoglobulin together with the tiny adapter reagent (Figure 3B–D).

## 3 Conclusions

While antibodies play an increasing role in medicine and life science research, the classical purification route via protein A affinity chromatography has remained the standard in many laboratories and the biopharmaceutical industry. In this work, we established an alternative approach for antibody purification based on a novel binding reagent derived from the archaebacterial protein Sac7d in combination with the Azo‐tag and light‐controlled α‐CD affinity chromatography.

X‐ray structural analysis revealed the mode of complex formation between the affitin Sac7d‐C3A24 and the IgG1 Fc fragment. Interestingly, each affitin molecule binds to one Fc chain at the junction between the C_H_2 and C_H_3 domains. In this manner, its binding site precisely overlaps with the ones of protein A and protein G, despite exhibiting a different protein fold. As a feature that was not expected from the engineering of the affitin,^[^ ^16^ ^]^ its C‐terminal residues are in direct contact with the Fc fragment and participate in a hydrogen bond network. Although the C‐terminal residue Lys66 seems to form a salt bridge with Lys248, SPR measurements of corresponding mutants indicated that an intramolecular H‐bond between Lys66 and the backbone oxygen of Glu62 may be even more important to stabilize the complex, as both a deletion of Lys66 and the substitution Lys66 → Ala resulted in a 2‐3‐fold reduced affinity. On the other hand, the N‐terminus of C3A24 seems not to be involved in interactions with the Fc fragment and, thus, should permit the construction of fusion proteins with other functional modules for applications in antibody research and technology.

An open question at the onset of this study related to the role of the single unpaired Cys residue at position 24, close to the center of the presumed binding site of the initially described affitin Sac7d‐C3.^[^ ^16^ ^]^ To prevent the formation of dimers via an intermolecular disulfide bridge, the authors proposed the replacement Cys24 → Ser, which also allowed the elucidation of the crystal structure as an isolated protein. However, C3(C24S) showed a 3‐fold reduced affinity compared to C3 in their measurements, rendering this mutant less suitable for application in affinity chromatography. Unexpectedly, the alternative side chain substitution Cys24 → Ala led to an even higher affinity as measured for C3 under the same experimental conditions in our hands, in line with its good sterical fit as evident from the present crystallographic analysis.

The successful use of the optimized monomeric affitin C3A24 for the construction of an adapter protein carrying the Azo‐tag has opened an innovative strategy for antibody purification in biomedical research. While the one‐step isolation of trastuzumab and rituximab from a cell culture medium with a high content of albumin relates to examples of clinically relevant antibodies, the light‐controlled affinity purification of recFc from the same type of crude matrix suggests broad applicability to all kinds of human IgG1 antibodies. Using just a short exposure to mild UV light for elution, common challenges associated with protein A/G affinity chromatography, such as the obligatory acidic pH shift during elution, which may cause antibody aggregation, Asn deamidation/isomerization and reduced yields, are circumvented.^[^ ^11a^ ^]^

In fact, the isocratic elution enabled by UV light preserves both the functional integrity of the antibody and of the column matrix, which can be reused many times. Furthermore, there is freedom of choice regarding the buffer, which allows direct use of the eluted antibody for subsequent screening experiments, such as affinity measurements or cell culture assays, without the need for buffer exchange. While the isolated antibody still contains the affitin adapter molecule as a minor impurity, this is acceptable for such functional studies considering that its binding site is far remote from the complementarity‐determining regions as well as from interfaces with Fcγ receptors and complement.^[^ ^41^ ^]^ In cases where the affitin adapter needs to be removed, for example, for animal studies with the purified antibody, this is easily achieved by SEC in the presence of a low concentration of urea at physiological pH (Figure 3E; Figure S4, Supporting Information). While the mAb·affitin complex is efficiently dissociated, antibodies are resistant to urea under such mild conditions.^[^ ^42^ ^]^ Apart from using C3A24—in Azo‐tagged form—as a promising alternative for the light‐controlled affinity purification of antibodies, the construction of N‐terminal fusion proteins, for example with enzymes or fluorescent proteins, may open additional applications for this affitin in antibody research.

## 4 Experimental Section

4.1

4.1.1

##### Vector Construction

The coding region of the human IgG1 Fc fragment derived from residues 236–444 of the rituximab heavy chain (PDB ID: 1L6X)^[^ ^43^ ^]^ was equipped with an N‐terminal His_6_‐tag and obtained as a synthetic gene with codons optimized for *E. coli* (GeneArt/Thermo Fisher Scientific, St. Leon‐Rot, Germany). The DNA fragment was subcloned onto the vector pASK111^[^ ^44^ ^]^ via the *Nde*I and *Hin*dIII restriction sites, and its sequence was confirmed by Sanger sequencing (Eurofins Genomics, Ebersberg, Germany).

Likewise, the coding region for the affitin Sac7d‐C3^[^ ^16^ ^]^ was obtained by gene synthesis with codons optimized for *E. coli* (GeneArt/Thermo Fisher Scientific), subsequently mutated and finally subcloned on a derivative of the expression plasmid pASK75^[^ ^45^ ^]^ harboring the gene for the mature MBP (UniProt ID: P0AEX9). The resulting fusion protein comprised the MBP, a His_6_‐tag, a TEV cleavable linker (ENLYFQ↓DAEF), and the affitin C3A24 starting at its residue Val2.

For affinity measurements and application of the affitin as an adapter protein in α‐CD affinity chromatography,^[^ ^23^ ^]^ the coding region for C3A24 was modified via PCR to incorporate an *Nde*I restriction site and an Azo‐tag (Gly‐Pap‐Gly‐Pro) at the N‐terminus, together with a *Hin*dIII restriction site at the C‐terminus, using the primers 5'‐AGTTTCATATGGGATAGGGTCCGGTCAAAGTAAAGTTTCTCTTGAATGGTGAGG and 5'‐CGAAGGAAGCTTACTTCTTCTCACGCTCCG. The resulting PCR product was digested with the two restriction enzymes and inserted into the expression vector pSB22‐PapRS#34, which encodes a Pap‐specific aminoacyl‐tRNA synthetase (PapRS#34) and the cognate suppressor tRNA^Pyl^.^[^ ^23^ ^]^ This single‐plasmid system enables the incorporation of the non‐canonical amino acid Pap via amber stop codon suppression. Compared with the previously published vector pSB19‐PapRS#34,^[^ ^23^ ^]^ two duplicate DNA segments were removed in order to improve the genetic stability of the plasmid: a short copied sequence stretch from the *lac*I promoter region close to the artificial *pyl*T gene (as remnant from previous cloning steps) was deleted, and a second copy of the *rrn*C terminator at the end of the coding region for PapRS#34 was replaced by the potent phage fd terminator.^[^ ^46^ ^]^ Amino acid exchanges in the encoded affitin were generated using the QuikChange site‐directed mutagenesis procedure (Agilent, Santa Clara, CA).

##### Protein Production and Purification

The recombinant Fc fragment (recFc) was produced in the cytoplasm of the *E. coli* strain Origami B (Merck Millipore, Darmstadt, Germany) after transformation with pASK111‐His_6_Fc. A bacterial culture in 2 L terrific broth (TB) supplemented with 100 mg L^−1^ chloramphenicol (Cam) was grown in a 5 L shake flask at 30 °C. At a cell density OD_550_ = 1.0, the *tet* ^p/o^ was induced by adding 100 μg L^−1^ anhydrotetracycline (aTc).^[^ ^45^ ^]^ After 4 h gene expression, the bacteria were harvested by centrifugation, resuspended in IMAC running buffer (50 mM Tris/HCl pH 7.5, 150 mM NaCl), and lysed using a PandaPLUS 1000 homogenizer (GEA, Düsseldorf, Germany). The recombinant protein was purified on a Ni‐NTA column (5 mL HisTrap HP; Cytiva, Freiburg, Germany) and eluted with a concentration gradient of 0–300 mM imidazole/HCl in IMAC running buffer. The remaining impurities were removed by CEX on a Resource S column (Cytiva) with 50 mM MES/NaOH pH 6.5 as running buffer using a concentration gradient of 0–500 mM NaCl in the same buffer for elution.

For the production of the affitin as a fusion protein, competent *E. coli* NEBExpress (New England Biolabs, Frankfurt/M., Germany) cells were transformed with pASK75‐MBP‐C3A24. A 2 L culture of Luria–Bertani (LB) medium supplemented with 100 mg L^−1^ ampicillin was grown at 30 °C to OD_550_ = 0.8. Protein expression was induced by adding 200 μg L^−1^ aTc, and the culture was incubated overnight at 30 °C. The bacteria were centrifuged, resuspended in IMAC running buffer pH 8 and mechanically lysed as above. After purification of the fusion protein from the lysate via IMAC (at pH 8), the resulting protein solution was supplemented with 5 mM DTT. Then, 0.38 μg His_6_‐tagged TEV protease, produced in house using the pRK793 plasmid^[^ ^47^ ^]^—gift from David Waugh (#8827; Addgene, Watertown, MA)—was added per 100 μg substrate. Proteolytic cleavage of the MBP fusion protein was accomplished by incubation overnight at 25 °C. Finally, subtractive IMAC was performed to recover the cleaved affitin, which carried the additional N‐terminal residues DAEF.

The affitin carrying an N‐terminal Azo‐tag was produced in *E. coli* NEBExpress(lowRF1) transformed with the expression vector pSB22‐PapRS#34 following a published procedure.^[^ ^23^ ^]^ Briefly, after inoculating 100 mL LB/Amp medium, the bacteria were grown to OD_550_ = 0.4. Then, 0.2 mM L‐Pap and 0.2% w/v L‐arabinose (from a stock solution containing 2 mM L‐Pap, 8 mM (2‐hydroxypropyl)‐β‐cyclodextrin and 2% w/v L‐arabinose in 0.2 M NaOH) were added. After 1 h, recombinant gene expression was induced by further adding 0.5 mM isopropyl‐β‐D‐thiogalactopyranoside (IPTG; from a 10 mM stock solution) and continued overnight at 30 °C. Bacteria were harvested by centrifugation, resuspended in α‐CD buffer (50 mM Tris/HCl pH 7.5, 150 mM NaCl), and lysed using a Digital Sonifier (Branson Ultrasonics, Danbury, CT). One‐step purification of the Azo‐tagged affitin was achieved by light‐controlled α‐CD affinity chromatography on a column with 1 mL bed volume as described.^[^ ^23^ ^]^ To this end, the sample (up to 5 mL bacterial extract) and washing steps (3 × 1 mL) were applied in the dark, followed by isocratic elution (2 mL) under illumination with LED UV light at 355 nm.

##### Isolation of the Affitin Sac7d‐C3A24 in Complex with a Human IgG1 Fc Fragment

Both purified proteins were separately concentrated to a final volume of 1.5 mL (recFc: 443 μM; C3A24: 1006 μM) using Amicon Ultra‐15 centrifugal filter units (3 kDa MWCO; Merck Millipore, Darmstadt, Germany). Protein concentrations were calculated according to the Lambert–Beer Law using the absorption at 280 nm with molar extinction coefficients *ε*_280_ = 70,820 L mol^−1^ cm^−1^ for recFc and *ε*_280_ = 2,980 L mol^−1^ cm^−1^ for C3A24 as well as corresponding molecular masses of 49,469.92 g mol^−1^ and 7,872.89 g mol^−1^, respectively. Both proteins were mixed at a stoichiometric ratio of 1:4 with respect to one recFc heavy chain and the affitin, followed by incubation at room temperature overnight. The complex was isolated using SEC on a Superdex 75 16/60 column (Cytiva) in the presence of α‐CD buffer. Fractions containing both the affitin and recFc were identified by SDS‐PAGE and pooled, yielding 5.8 mg protein. After buffer exchange to 50 mM Tris/HCl pH 7.5 by dialysis, the complex was concentrated to 8.1 mg mL^−1^ and sterile‐filtered using Ultrafree Centrifugal Filters PVDF 0.22 μm (Merck Millipore).

##### Protein Crystallization and X‐ray Structure Determination

The recFc·affitin complex was crystallized at 20 °C using the hanging drop vapor diffusion technique and an in‐house precipitant screen. The final droplets comprised 2 μL distilled water (to slow down equilibration), 1 μL protein solution, and 1 μL precipitant solution containing 24% w/v PEG8000, 0.1 M Tris/HCl pH 8.5. Crystals were harvested, transferred to the precipitant buffer supplemented with 25% v/v glycerol as cryoprotectant, and immediately frozen in liquid nitrogen. X‐ray diffraction data were collected at beamline P13 (PETRA III) of the DESY synchrotron (Hamburg, Germany). The complex crystallized in the space group P2_1_ (Table 1), with 2 biological protein complexes (2 homodimeric Fc fragments, 4 affitin molecules) in the a.u.

X‐ray diffraction data were processed and scaled with the XDS software package.^[^ ^48^ ^]^ Molecular replacement was carried out with Phaser from the CCP4 program suite^[^ ^49^ ^]^ using the coordinates of the closely related individually crystallized affitin Sac7d‐C3(C24S) (PDB ID: 2XIW)^[^ ^16^ ^]^ and the recombinant human IgG1 Fc fragment of rituximab (PDB ID: 1L6X)^[^ ^43^ ^]^ as search models. The atomic model was built with Coot^[^ ^50^ ^]^ and refined with Refmac5,^[^ ^51^ ^]^ including correction using the PDB_REDO server.^[^ ^52^ ^]^ The refined structural model was validated with PROVE,^[^ ^53^ ^]^ ERRAT,^[^ ^54^ ^]^ Verify3D,^[^ ^55^ ^]^ PROCHECK,^[^ ^56^ ^]^ WHAT_CHECK,^[^ ^57^ ^]^ and via the MolProbity server.^[^ ^58^ ^]^ Secondary structures were assigned using DSSP,^[^ ^59^ ^]^ and protein/ligand contact surfaces were calculated with PISA.^[^ ^34^ ^]^ Molecular graphics were prepared using PyMOL software (Schrödinger, New York, NY).

The Fc·affitin complex revealed electron density for 1112 of in total 1144 protein residues present in the a.u., namely Gly^236^–Ser^444^ of all copies of recFc (chains A, B, C, D) and Asp^−2^–Lys^66^ of the four C3A24 molecules (chains R, S, T, U). The closely associated chains A and R within the a.u. were selected for further structural analysis based on the comparably high quality of their electron density maps. The atomic coordinates and structure factors have been deposited at the Protein Data Bank (PDB), Research Collaboratory for Structural Bioinformatics (Rutgers University, New Brunswick, NJ), under accession code 9HIZ.

##### Affinity Measurements

Real‐time SPR measurements were performed on a BIAcore X100 system (GE Healthcare, Uppsala, Sweden) using HBS/T (20 mM Hepes/NaOH pH 7.4, 150 mM NaCl, 0.005% v/v Tween‐20) as running buffer (flow rate = 25 μL min^−1^) at 25 °C. Herceptin (Roche, Grenzach‐Wyhlen, Germany) was immobilized onto a carboxymethyl dextran‐coated CM3 sensor chip (Cytiva) via amine coupling chemistry to achieve a resonance response ΔRU ≈ 2000. A 2:1 dilution series of the purified Azo‐tagged affitin solution was injected as analyte using single‐cycle kinetics.^[^ ^60^ ^]^ Due to the very low absorbance of the affitin at 280 nm, the protein concentration was calculated using the specific absorbance of the Pap residue at 326 nm. First, to account for the *cis*/*trans* ratio of Pap at the photostationary state under daylight, the protein solution was illuminated for 5 min with LED light at a wavelength of 430 nm. Then, absorption spectra were recorded, and the A_326_/A_426_ ratio was used to calculate the molar extinction coefficient at 326 nm with the formula described by Mayrhofer et al.^[^ ^23^ ^]^ The raw SPR data were corrected by subtracting 1) the corresponding signals measured for the control channel and 2) an averaged baseline determined from three buffer blank injections.^[^ ^61^ ^]^ The resulting sensorgrams were analyzed with the Biacore X100 Evaluation Software (ver. 2.0.1). Dissociation constants (*K*_D_) were obtained by fitting the response values at equilibrium using a steady‐state affinity analysis for three separate measurements. To determine the rate constants of association and dissociation, *k*_on_ and *k*_off_, a two‐state reaction model was used that also accounted for a secondary conformational change.

##### Light‐Controlled α‐CD Affinity Purification of IgG1 Antibodies from Cell Culture Medium Using an Affitin‐Based Adapter Molecule

Azo‐C3A24 was purified by α‐CD affinity chromatography as described further above, followed by exposure to 430 nm light for 15 min to revert the Pap residue to the predominant trans‐configuration. For the antibody purification experiments, 800 μL Dulbecco's Modified Eagle's Medium (DMEM) containing 5% v/v fetal calf serum (FCS) was spiked either with 0.1 mg Kanjinti (Amgen, Thousand Oaks, CA), 0.1 mg MabThera (Roche, Grenzach‐Wyhlen, Germany), or 0.03 mg recFc from above. These solutions were mixed with the fourfold molar equivalent of Azo‐C3A24, incubated either for 10 min or overnight at room temperature, and centrifuged (3 min, 20,000 × g). The clear protein solution was applied to the α‐CD affinity column (1 mL bed volume), followed by washing with 3 mL α‐CD buffer. Bound protein was eluted in 2 mL α‐CD buffer under exposure to 355 nm UV light. To remove the adapter molecule, a mixture of purified trastuzumab and Azo‐C3A24 in α‐CD buffer was supplemented with 1.5 M urea (from a stock solution of 7.5 M in α‐CD buffer) in a total volume of 150 μL, incubated for 45 min at room temperature, and applied to an analytical SEC column (Superdex S200, 10/300 GL Increase; Cytiva) using a running buffer consisting of α‐CD buffer and 1.5 M urea.

##### Protein Analytics

SDS‐PAGE was performed using a published buffer system^[^ ^62^ ^]^ and staining with Coomassie brilliant blue. Mass spectra of proteins were measured on an impact II time‐of‐flight (TOF) mass spectrometer with an electrospray ionization (ESI) source (Bruker Daltonics, Bremen, Germany) in the positive ion mode. To this end, the purified protein was dialyzed against 10 mM ammonium acetate pH 6.6, followed by the addition of 50% v/v methanol and 1% v/v formic acid, and directly applied to the instrument via a syringe pump at 90 μL h^−1^. Raw spectra were deconvoluted with the Bruker Compass Data Analysis Software using the MaxEnt algorithm.^[^ ^63^ ^]^

## Conflict of Interest

The authors declare no conflict of interest.

## Supporting information

Supplementary Material

## Data Availability

The data that support the findings of this study are openly available in Protein Data Bank at https://www.rcsb.org/structure/unreleased/9HIZ, reference number 0.
