# Supplementary material for: Engineering and Structural Elucidation of a Sac7d‐Derived IgG Fc‐Specific Affitin and Its Application for the Light‐Controlled Affinity Purification of Antibodies
Source: Chembiochem. 2025 Apr 7;26(11):e202500102. doi: 10.1002/cbic.202500102 (PMC12135134; doi:10.1002/cbic.202500102)
Supplement: Supplementary file 1 — Supplementary Material [file CBIC-26-e202500102-s001.pdf]

Engineering and structural elucidation of a Sac7d-derived IgG Fc-specific affitin and its application for the light-controlled affinity purification of antibodies

Felix Veitl, Andreas Eichinger, Peter Mayrhofer, Markus R. Anneser, Mauricio Testanera, Kilian Rauscher, Matthias Lenz, Arne Skerra*

Chair of Biological Chemistry, School of Life Sciences, Technical University of Munich, 85354 Freising, Germany

*Corresponding author:

Prof. Dr. Arne Skerra; phone: +49 8161 71 4351; fax: +49 8161 71 4352;

e-mail: skerra@tum.de

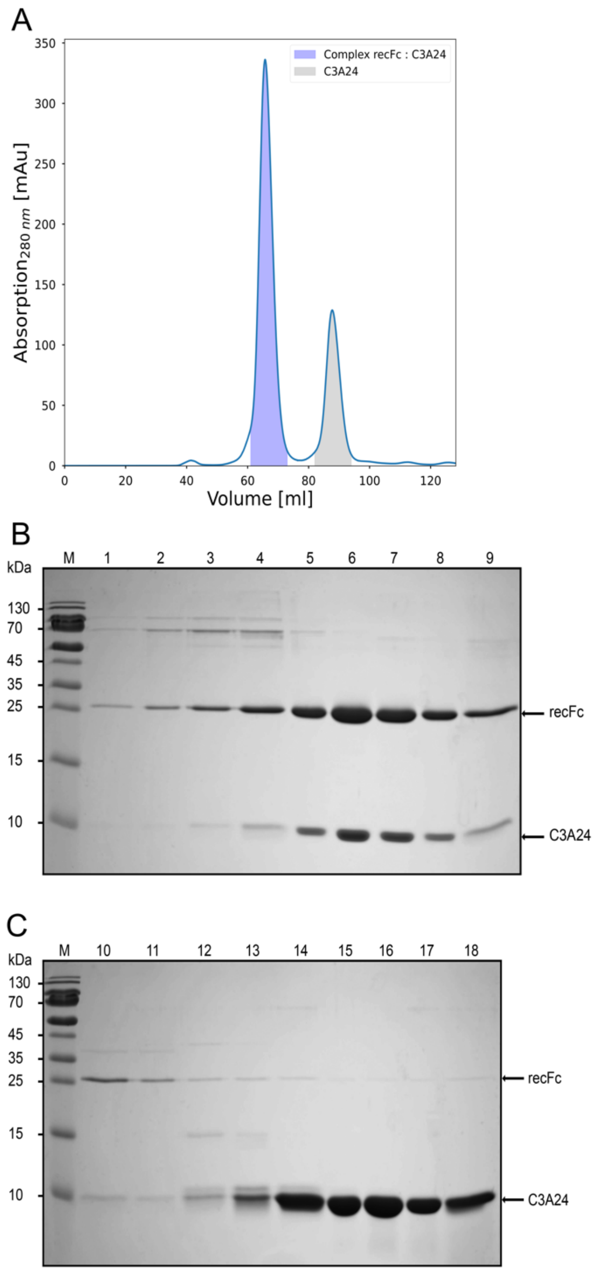

Suppl. Figure S1. Isolation of the recFc•C3A24 complex. (A) SEC chromatogram of the mixture of both purified protein components in 50 mM Tris/HCl pH 7.5, 150 mM NaCl after incubation overnight. The area under the curve containing the complex (blue) and excess affitin (grey) has been colored according to the SDS-PAGE analysis (B, C). Lane 1: PageRuler Prestained Protein Ladder; lanes 2-18: consecutive SEC fractions.

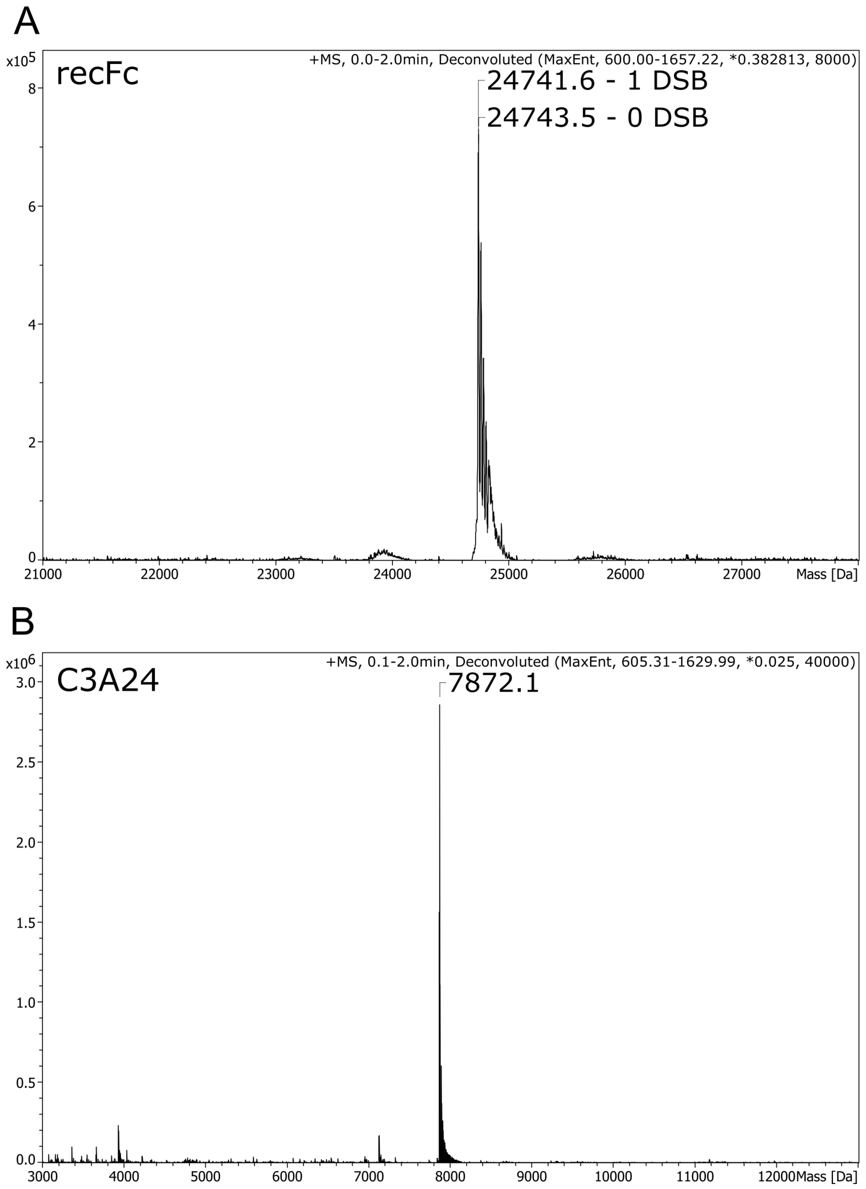

Suppl. Figure S2. Mass spectrometric analysis of the proteins used for the crystallographic analysis. (A) Deconvoluted ESI-MS of recFc: M_{calc} = 24,739.97 Da (one Fc heavy chain with disulfide bonds); $M_{obs\#1}$ = 24,743.6 Da, ΔM = +4 Da (no disulfide bond); $M_{obs\#2}$ = 24,741.6 Da, ΔM = +2 Da (one disulfide bond). (B) Deconvoluted ESI-MS of C3A24 (carrying the additional N-terminal residues DAEF): M_{calc} = 7,872.89 Da; M_{obs} = 7,872.1 Da.

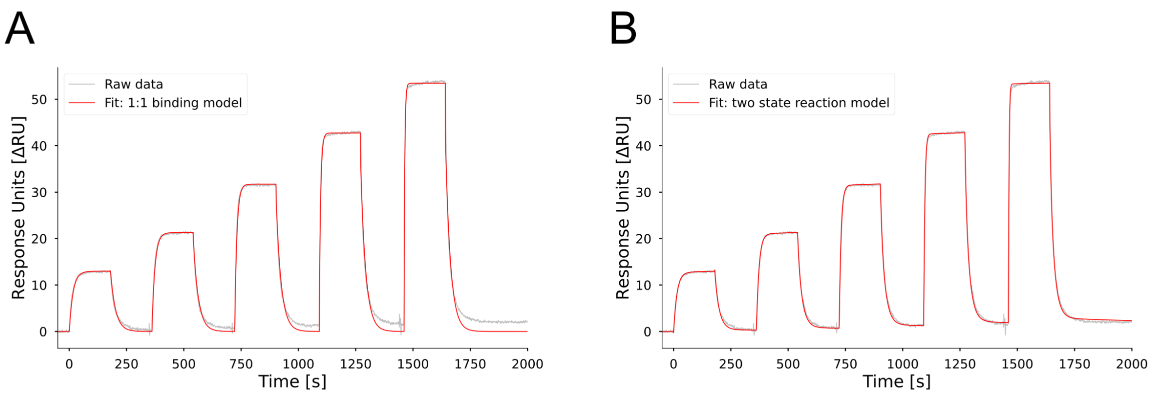

Suppl. Figure S3. Affinity measurements using real-time SPR spectroscopy and a sensorchip coated with trastuzumab. Sensorgrams obtained for Azo-C3A24 and fitted according to a 1:1 binding model (A) or to a two-state reaction model (B).

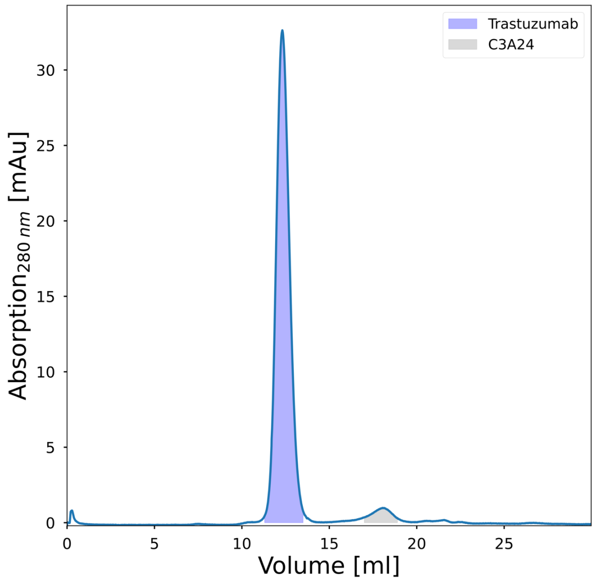

Suppl Figure S4. Separation of trastuzumab from Azo-C3A24. To achieve dissociation, the trastuzumab•affitin complex was incubated for 45 min in 50 mM Tris/HCl at pH 7.5, 150 mM NaCl after addition of 1.5 M urea and then subjected to SEC on a Superdex S200, 10/300GL Increase column using the same running buffer. The antibody elutes in a distinct peak in pure state whereas the much smaller affitin is strongly retarded.

Suppl. Table S1. Kinetic constants determined by SPR spectroscopy for affitins *versus* trastuzumab after data fit according to a two-state reaction model.

	$k_{on\#1}$ [1/M×s]	$k_{off\#1}$ [1/s]	$k_{on\#2}$ [1/s]	$k_{off\#2}$ [1/s]
**C3A24**	$1.64 \pm 0.34\times10^{6}$	$9.38 \pm 3.2\times10^{-2}$	$1.64 \pm 2.4\times10^{-3}$	$9.64 \pm 16\times10^{-3}$
**C3A24(ΔK66)**	$9.12 \pm 3.8\times10^{5}$	$1.73 \pm 0.54 \times10^{-2}$	$2.34 \pm 2.0\times10^{-4}$	$9.48 \pm 15\times10^{-4}$
